# Students under lockdown: Comparisons of students’ social networks and mental health before and during the COVID-19 crisis in Switzerland

**DOI:** 10.1371/journal.pone.0236337

**Published:** 2020-07-23

**Authors:** Timon Elmer, Kieran Mepham, Christoph Stadtfeld

**Affiliations:** Social Networks Lab, Department of Humanities, Social and Political Sciences, ETH Zürich, Zürich, Switzerland; Middlesex University, UNITED KINGDOM

## Abstract

This study investigates students’ social networks and mental health before and at the time of the COVID-19 pandemic in April 2020, using longitudinal data collected since 2018. We analyze change on multiple dimensions of social networks (interaction, friendship, social support, co-studying) and mental health indicators (depression, anxiety, stress, loneliness) within two cohorts of Swiss undergraduate students experiencing the crisis (N = 212), and make additional comparisons to an earlier cohort which did not experience the crisis (N = 54). In within-person comparisons we find that interaction and co-studying networks had become sparser, and more students were studying alone. Furthermore, students’ levels of stress, anxiety, loneliness, and depressive symptoms got worse, compared to measures before the crisis. Stressors shifted from fears of missing out on social life to worries about health, family, friends, and their future. Exploratory analyses suggest that COVID-19 specific worries, isolation in social networks, lack of interaction and emotional support, and physical isolation were associated with negative mental health trajectories. Female students appeared to have worse mental health trajectories when controlling for different levels of social integration and COVID-19 related stressors. As universities and researchers discuss future strategies on how to combine on-site teaching with online courses, our results indicate the importance of considering social contacts in students’ mental health and offer starting points to identify and support students at higher risk of social isolation and negative psychological effects during the COVID-19 pandemic.

## Introduction

The COVID-19 pandemic has forced leaders in politics and at universities to take drastic measures that affect how citizens and students interact and socialize with each other. In many countries around the world, individuals are required to reduce physical contact to others outside one’s household (*social distancing*) [1]. Additional measures include curfews, quarantines, and closing of non-essential stores, schools, and universities. As many universities suspended classroom teaching and switched to online teaching, the lives of students have changed drastically. While social distancing measures may successfully slow down the spread of the infection and relieve the public health systems [2], they may eventually increase the social isolation of students and affect their psychological well-being and mental health [3]. Being under a lot of pressure to perform academically, students are prone to developing mental health problems [4]. The social networks of students have been argued to be an important factor in buffering stress and helping them to be more effective [5]. Reduced social interactions, a lack of social support, and newly arising stressors associated with the COVID-19 crisis could potentially affect students’ mental health negatively. In line with other ongoing research studies conducted across the globe, we examine the effects of the COVID-19 crisis on a student population, e.g., [6–9]. At the time of the final data collection (early April 2020), their university had switched to online teaching, university staff was working from home, and all buildings were closed. In Switzerland, non-essential shops were closed, and social gatherings were restricted to groups of at most five people. Furthermore, in-person socializing with others beyond co-residents was advised against. We ask two focal questions: how the COVID-19 crisis affects the social networks between students, and how changes in students’ social networks and daily lives affect students’ mental health (i.e., depressive symptoms, anxiety, loneliness, and stress).

### Empirical setting

**Fig 1:**
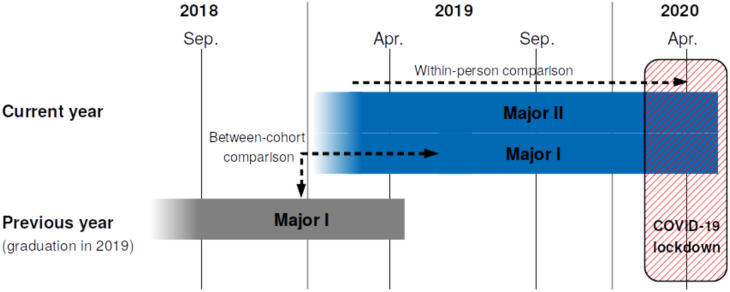
Timing of social network and mental health data collection, and empirical comparisons. Students were enrolled in one of two study majors and one of two year groups.

The timing of the data collection relative to the COVID-19 crisis and the empirical comparisons are illustrated in Fig 1. Social networks and mental health outcomes are investigated from 2018 until the time of the COVID-19 crisis in April 2020, two weeks after Switzerland introduced social distancing measures and a strict university lockdown was put into place. We examine changes in social integration and mental health through two lenses: within-person, using data collected during the two years prior to the COVID-19 crisis, and between-cohort, using data collected in one cohort of students in an undergraduate program a year earlier. Integration in social networks is examined *within* the communities of students and *beyond*, including social contacts in the household, family, and friendship circles.

We investigate the longitudinal associations between social networks and mental health markers with three pre-registered hypotheses predicting decreased social integration, higher survival chance of stronger, overlapping social ties, and mixed effects of the crisis on mental health (osf.io/4g98p). Furthermore, we explore individual and social factors associated with change in mental health during the COVID-19 crisis.

### Changes in social networks

The social relationships of individuals are likely to be affected by the crisis in different ways. Social relationships are conduits of social support [10]. In times of crisis, social support may be more important than ever. But at the same time, physical proximity and opportunities for interaction are important in developing and fostering social ties [11–13]. As face-to-face interactions and random encounters are minimized due to the social distancing measures, it is likely that individuals focus on those relationships that are spatially close, most meaningful, or most established. Those may partly be found outside the student community, for example, in the household, in the family, and within established friendship circles [14].

In this article, we take into account multiple dimensions of social relations. We assess change in five self-reported social networks within the student community: pleasant interactions, friendships, emotional support, informational support, and co-studying. We chose those networks as they differ in function, intimacy, and stability. We assume that stronger relationships will be characterized by an overlap in multiple dimensions [15].

We hypothesize that students at the time of the COVID-19 crisis *nominate fewer fellow students* than before in multiple dimensions of their social networks (*H1a*). We expect that social networks between students are less connected, and thus will show a *higher rate of students being socially isolated* (*H1b*). We hypothesize that *stronger ties are more likely to survive* at the time of crisis, thus when they are characterized by a previous overlap in multiple relational dimensions (*H2*).

### Changes in mental health

Prior research conducted with individuals in strict quarantine suggests severe consequences for mental health, for a review see [16]. It is unclear, however, to what extent these findings are comparable to the less severe distancing measures implemented under COVID-19.

Changes in social networks due to the COVID-19 crisis may directly affect individuals’ mental health. Most individuals have a fundamental motivation to socially interact [17]. A reduction of social interactions, as is likely to be caused by the crisis and distancing measures, can lead to lower mental health [18, 19].

A number of COVID-19-specific stressors could further affect individuals’ mental health. Among those are worries about one’s own physical health, the health of others, the potential economic impact, and—in the case of students—the impact of a changed educational environment on the progress of their studies and future job market opportunities. However, some individual stressors may be reduced at the time of crisis, as society slows down [20]. Those may, for example, include the stress of work, such as rigid study time tables, or social stressors such as Fear of Missing Out (FoMO) [21].

We expect that during the COVID-19 crisis, *some aspects of students’ mental health will improve* (e.g., aspects of daily stress) *while others will worsen* (e.g., loneliness) (*H3*). To get a better understanding of factors explaining change in mental health, we further *explore the impact of different individual and social factors on change in mental health*. We consider COVID-19-related stressors, social network integration within the student community, social ties outside the student community, and demographic factors. The identified individual and social factors could be relevant starting points for future empirical studies and could inform potential interventions to combat the effects of the COVID-19 pandemic on social isolation and individuals’ mental health.

## Methods

### Participants

The participants were all students, starting their undergraduate studies in 2017, in two different engineering / natural science programs (henceforth referred to as current year, Major I and Major II), and students starting in 2016 in one study program (previous year). Students in the previous-year cohort followed the same program as the current year, Major I (see Fig 1).

In the current-year cohort, participants are selected on response to both of two questionnaires in April 2020 (during the COVID-19 lockdown) and September 2019, while the sample from the previous-year cohort is selected from those responding to a questionnaire in April 2019. All students that were enrolled in the study program of the respective cohorts were invited to participate in the surveys. Participants were excluded from the data if they started but did not complete the survey (*N_Sept_* = 15, *N_April_* = 12), failed the attention check item (i.e., “To ensure that your web browser is functioning correctly, please click on option 4 [out of 6]”; *N_Sept_* = 10, *N_April_* = 14), or responded to the whole questionnaire in less than 15 minutes (*N_Sept_* = 2, *N_April_* = 1; median response time: 49 and 50 minutes, respectively). The sizes of the samples used in our analyses are respectively *N_1_* = 86, *N_2_* = 196, *N_3_* = 54. Depending on the test employed, we subset on those individuals in the current-year cohort for whom we have repeated observations, and individuals in the same major. All samples have a higher proportion of male students, and a majority of Swiss nationals (see Table 1).

**Table 1:** Demographics of each sample.

Cohort	*N*	*N*_repeat_	% Women	(% miss.)	% Swiss	(% miss.)
Current year, Major I	86	70	33.7	(10.5)	73.3	(–)
Current year, Major II	196	142	15.3	(2.0)	89.8	(–)
Previous year, Major I	54	–	38.9	(–)	74.1	(9.3)

*N* indicates those who responded to the April 2020 survey (or April 2019 for the previous-year cohort). The demographic percentages are based on this *N*. *N_repeat_* indicates the number of individuals who responded to both in the April 2020 and September 2019 surveys. Miss. = missings

### Procedure

Data were collected (a) prior to the COVID-19 outbreak as part of the Swiss StudentLife study (September 2017 to September 2019) [5], and (b) two weeks after the university and Swiss public life were largely shut down in response to the crisis (April 2020). A comparable survey was administered in the previous year cohort who had started their undergraduate studies one year earlier but did not experience this lockdown (previous year cohort; April 2019). The April surveys took place about half-way through the Spring semester (the final semester for the undergraduate students in the sample; February—May), the September surveys at the beginning of the Autumn semester (September—December). For the supplementary trend analyses reported in the S1 Appendix, we use all comparable data from Major I of both cohorts, collected over ten waves of the Swiss StudentLife Study spanning September 2016-2019 (for the previous year cohort) and 2017-2020 (for the current year cohort). Each round of surveys was spaced approximately 2 to 3 months apart.

Surveys were conducted online using the Qualtrics software. The students were invited by email and SMS with personalized links, and informed of the compensation of 30 Swiss Francs per survey (approximately 31 USD or 28.5 EUR in April 2020). The amount equals the compensation for participation in one hour of experimental research at the same institution. Each questionnaire was open for one week, within which students could respond at their convenience. In this period, students were reminded to participate twice. The questionnaire was administered in German, the language of instruction at the Swiss university where the study was conducted.

The first two pages of the questionnaire always stated the purposes and uses of the data, and asked for consent to these. The students were asked if they would like to receive an information email if their mental health as measured by psychometric scales was above a threshold indicative of a potential clinical relevance. After this, they filled out the questionnaire, lasting a median of 49 and 50 minutes for the current year cohort in September 2019 and April 2020, and 58 minutes for the previous year cohort in April 2019. The questionnaire in April 2020 was reduced in terms of content (with a small number of COVID-19 related items added), but the ordering and formulation of questions remained identical. The data collection and intended uses were approved by the institutional ethics committee of ETH Zürich (approval numbers 2016-N-27 and 2017-N-42).

### Materials and variable construction

#### Cohort network items

To explore changes on complete social networks, various questions about individuals’ relations to others within the cohort were asked. Focal relations include perceived pleasant interactions with other students (in the following: Interaction), friendships, co-studying, reception of informational support, and reception of emotional support. Furthermore, participants were asked about their communication with others in the cohort; in particular with whom they had contact face-to-face (Physical communication), over messaging applications (Messaging), by video or audio (Calls), or by social media applications that do not fall under the previous categories (Social Media). The precise wording of these items is provided in the S1 Appendix. For all of these social network items, a name generator approach was applied [22]: participants could fill in up to 20 auto-completing text fields, in which names of all students within that cohort’s major program were listed. Each name reported was treated as a tie from the respondent to that individual within the respective network. The rate of mutual nomination, i.e. the relative frequency of two people nominating one another in the same network, is reported in the S1 Appendix.

All network items were included in both surveys for the current-year cohort, and for the survey in the previous-year cohort, with the exception of the physical and calls contact networks.

For each type of network captured by these items, we construct two key measures to test Hypotheses 1a and 1b: the *outdegree* of each respondent, i.e. the number of ties that each participant reports in each network, and the proportion of *out-isolates*, i.e. the fraction of all participants who reported no ties in a network. To test Hypothesis 2, we use the overlap of multiple ties within pairs of individuals collected in September 2019 as a signal of tie strength.

#### Personal network items

To examine individuals’ personal networks (ego networks) [23], respondents were asked about people who are important to them during the COVID-19 crisis. Here, participants could still name fellow students but also others, for example, a partner, family members, housemates, colleagues, or friends. Translated versions of all personal network items used in our analyses are available in the S1 Appendix.

Respondents were prompted to fill in pseudonyms that they would recognize for up to 10 individuals (*personal network size*). After indicating their names, respondents were asked a number of follow-up questions. They were asked about contexts in which they communicate with these individuals (six multiple-choice options): Within the household, outside of the household, by telephone, by FaceTime/Skype or similar, over messaging applications, or other (with an open text box to further specify the context). They were asked about the roles that these individuals fulfil (e.g., partner, family member, friend, etc.; multiple-choice), and whether they receive emotional support from each person on a 7-point ordinal scale, ranging from completely disagree (1) to completely agree (7). Additionally, participants were asked how often they communicated with these people in-person, by phone, or over messaging applications on a 7-point ordinal scale, ranging from 1 (not at all) to 7 (multiple times a day). These ego network items were only asked in the April 2020 questionnaire of the current year cohort.

#### COVID-19 items

A battery of items presented in the April 2020 wave captured the extent of participants’ experiences of stressors relative to the time before the COVID-19 crisis, on topics including one’s own health, family and friends, one’s personal finances, the economy, and one’s career prospects. Other items in this battery captured the extent to which respondents fear missing out on social activities, feel socially isolated, feel competition among students, feel supported by other students, and the extent to which they face personal problems they previously suppressed compared to the time before the crisis. All answer categories were on the same 7-point scale, ranging from -3 (much less than before) to 3 (much more than before). The midpoint of the scale was 0 (equally). Full item formulations are listed in the S1 Appendix.

Furthermore, specific to the temporal context under study, participants in the April 2020 questionnaire were asked questions pertaining to health and health guidelines. First, they were asked whether they have had any symptoms consistent with COVID-19 infection, which they answered with a multiple choice list of symptoms. Following this, participants were asked whether someone they are close to is in an at-risk group, with a yes/no response. They were additionally asked how strictly they are adhering to federal guidelines against the disease’s spread, using a five-point scale ranging from 1 (not at all) to 5 (always).

#### Mental health scales

*Depressive symptoms* were measured by the German version of the Center for Epidemiologic Studies Depression scale [24]. This measure comprises 20 items on a 4-point scale ranging from 0 (occurred never or rarely) to 3 (occurred often or always). Each item reflects the frequency of a symptom in the past week, with symptoms including “I felt that everything I did took a lot of effort”, and “I felt depressed”. Depression is scored as the sum of all items, thus ranging from 0 to 60. Cronbach’s alpha of the measure is *α* = .91.

*Anxiety* was measured by the German version of the Generalized Anxiety Disorder-7 scale [25, 26]. This 7-item measure has responses on a 4-point scale ranging from 0 (not at all) to 3 (nearly every day), regarding the frequency of experiencing each symptom in the past two weeks. Examples include having “trouble relaxing”, and “feeling afraid as if something awful was about to happen”. Anxiety is sum-scored from all the items—ranging from 0 to 21. Cronbach’s alpha of the measure is *α* = .88.

*Stress* was measured by the German version of the Perceived Stress Scale-10 [27]. This measure comprises 10 items on a 5-point scale, ranging from 0 (never) to 4 (very often), each capturing the frequency of a marker of stress over the past month. Although we aimed to capture the stress levels of the two weeks since the lockdown, asking about perceived stress within the past month was kept for comparability reasons with previous waves. Examples include how often the respondent felt “able to control irritations in your life”, or were “upset because of something that happened unexpectedly”. The stress score is computed as the sum-score of all items, therefore ranging from 0 to 40. Cronbach’s alpha of the measure is *α* = .86.

*Loneliness* was measured by the short, German version of the UCLA Loneliness Scale [28, 29]. This scale contains 9 items with responses on a 4-point scale ranging from 1 (Never) to 4 (Always), capturing how often respondents experience feelings of loneliness. Items include “How often do you feel that you lack companionship?” and “How often do you feel that there are people you can talk to?”. The loneliness score is computed as the mean score of all items, hence ranges from 1 to 4. Cronbach’s alpha of the measure is *α* = .86.

#### Extraversion

In our exploratory analysis on changes in mental health, we further control for the general sociability of individuals. We proxy this sociability with extraversion, measured by the 8-item subscale of the German version of the Big Five Inventory [30]. Responses for each item, capturing the extent to which a statement reflected the respondent’s self-perceptions, ranged from 1 (Very well) to 5 (Not at all). Examples of items include “I see myself as someone who is talkative” and “I see myself as someone who is full of energy”. The extraversion score was computed as the mean of the items. This measure had a Cronbach’s alpha of *α* = .86.

#### Factors for exploring changes in mental health

Multiple blocks of variables are included in an analysis of the change of the four mental health indicators from September 2019 until April 2020, to capture known and hypothesized stressors during the COVID-19 crisis. We include variables grouped into four blocks.

First, since concerns developing as a result of the virus may have implications for mental health, all items described above on COVID-19 related stressors crisis were included as potential factors. Two items on perceived additional stress and perceived additional relaxation were assessed but not used in the exploration of mental health changes, as those were identified as theoretically too close to the dependent variables.

Second, we identify those who did not report anyone (out-isolation) in the friendship, pleasant interactions, emotional support, informational support, and co-studying networks. These variables are included as social isolation (as indicated by being an isolated node in the network) may have negative individual consequences [31].

Third, personal network characteristics are included as covariates. We include the number of individuals named in the personal network questions (personal network size; ranging between zero and ten). Further, physical isolation in personal networks is captured by whether one is not living with someone who they indicate as being important since the lockdown (isolated household variable). We evaluate how often individuals have face-to-face contact with an important individual (minimal physical contact; measured as the maximum reported amount of face-to-face contact among all individuals in the personal network). Additionally, the mean level of emotional support provided by the individuals in the personal network is included (mean emotional support). Furthermore, two other specific COVID-19-related items were used to explore changes in mental health, specifically: at-risk status of close others and adherence to the federal health administration’s guidelines to prevent the spread of the disease.

Fourth, demographic and personal attributes were included to understand whether certain demographic categories or sub-populations might be particularly at risk beyond other factors: non-Swiss nationality, single relationship status, female gender, and extraversion.

Finally, covariates were included for all measures of mental health in September 2019 to prevent biases due to previous poor mental health. We additionally controlled for the study major, as the cohorts might differ in their general levels of mental health.

### Analysis

#### Assessing changes in social networks

To assess within-person changes on social networks, we compare the entire current-year cohort in April 2020 to the most recent previous observation (September 2019), selecting those individuals who responded to our survey at those timepoints (*N* = 212). The within-person comparisons of outdegree (H1a) and isolation proportions (H1b) of the observed network dimensions are used to test hypotheses one. The expected within-person decrease in outdegree between the two time points is examined by one-sided, paired-samples t-tests, with effect size given by Cohen’s *d* corrected for pairing [32]. The expected increase in proportion of out-isolates is examined within-group by McNemar’s test [33] with continuity correction to account for a low number of observations. Effect size is reported as the odds of transition from connected to isolated compared to isolated to connected, across the two time points. Further qualitative and structural differences in the network are examined through use of multiplex network visualizations. While this strategy allows for identification of changes, it does not distinguish between change due to general trends within the cohort, or changes brought about by the COVID-19 crisis and social distancing measures.

To build stronger evidence of the effects related to the COVID-19 crisis, we also compare all individuals in the current-year cohort, Major I who responded to the survey in April 2020 (*N* = 86), to individuals in the previous-year cohort who responded to our survey in April 2019 (*N* = 54) in between-cohort tests. We select only Major I of the current-year cohort for better comparability between cohorts, as the previous-year cohort followed the same major, and size and gender balance are closer to equal. Furthermore, this helps avoid potential effects of different majors such as different levels of stress related to specific majors or selection effects which could bias comparisons of absolute levels of mental health or social integration. Finally, we highlight that since these are between-cohort comparisons we do not need to take the subset of our current year, Major I cohort which responded to two questionnaires, but all of those who responded in April 2020 (*N* = 86).

To test the whether mean outdegree in each network is lower in the current year compared to the previous, we apply one-sided, independent-samples t-tests. These tests are Welch-corrected since group sizes are different and variance should thus not be assumed equal. To test the expected higher proportion of out-isolates in the current year compared to the previous, we use one-sided, two-sample *z*-tests [34], with Yates’ continuity correction to adjust for our relatively small sample. In both cases, effect size is given by Cohen’s *d*.

Additional testing of between-cohort difference allows better inference than from the within-person comparisons alone. However, despite matching the samples on their major program, there are nonetheless differences in baseline mental health, network structures, composition and time that limit our ability to infer causality. These differences are discussed in the results section and reported in the S1 Appendix.

To test Hypothesis 2, on the survival of social ties, we count the number of overlapping social network dimensions within each pair of individuals in September 2019. More specifically, we count the number of ties between two individuals in the given networks (pleasant interaction, friendship, co-studying, informational support, emotional support), excluding the target network (e.g., pleasant interactions). We then evaluate the survival rate of each number of overlapping dimensions (i.e., 0–4) relative to the baseline survival rate of the network. The baseline survival rate represents the proportion of, e.g., interaction ties, that were present in the September 2019 wave and the April 2019 wave *irrespective* of how many overlapping dimensions there were in September 2019. The survival rate of each category of overlapping ties is then computed solely on the subsample of which a given number of overlapping ties existed in September 2019. This way we can assess if having overlapping network dimensions in September 2019 contribute to the survival of ties until April 2020.

To obtain confidence intervals evaluating statistical significance, we apply bootstrapping [35] in the following way: For each number of overlapping dimensions, we sample (with replacement, i.e. bootstrapping) 3000 tie survival observations (0 = no tie, 1 = tie survived) of the observed target network (i.e., pleasant interactions / co-study). From this sampled distribution, the 95% confidence intervals around the observed proportion of ties that survived are computed.

#### Assessing changes in mental health

Similar to the assessment of changes in social networks, the changes in mental health are evaluated based on within-person and between-cohort comparisons. Since the preregistered hypotheses predicted changes in mental health but did not precisely record the direction of change for each variable, these comparisons are all conducted with two-tailed tests. The within-person comparisons are carried out using two-sided paired *t*-tests comparing the April 2020 assessments of mental health dimensions to those of September 2019. Here we again select our sample on two criteria: being in the current-year cohort, and observed at both timepoints (*N* = 212).

In the between-cohort comparison, mental health dimensions of the current-year cohort, Major I in April 2020 (*N* = 86), are compared to the measures of the previous-year cohort, assessed in April 2019 (*N* = 54). These between-cohort comparisons are made with two-sided *t*-tests, and are carried out to provide better evidence of whether changes in mental health are linked to the social distancing measures, rather than general study-progress related trends. Nevertheless, we note that it might not be valid to compare these two cohorts to one another due to prior differences in the general trends (see S1 Appendix).

Furthermore, the items on COVID-19 related stressors (see the S1 Appendix) are examined descriptively, in cross-section. Together with the overall mental health changes, these items on COVID-19 stressors are used to test Hypothesis 3.

#### Predictors of change in mental health

The relative importance of factors relating to COVID-19 specific stressors, student networks, personal networks, and demographic variables is explored using linear regression models. The dependent variables are the individual differences between the September 2019 and the April 2020 wave on the four mental health dimensions. We thus examine all current-year cohort members who responded to the relevant parts of our surveys at these timepoints (*N = 212*, reduced to *N* = 144 to 178, using listwise deletion). Positive values in the dependent variables indicate that the April 2020 scores were higher than those of September 2019. Given the high number of candidate variables relative to the sample size, we apply stepwise backwards selection procedure to preserve statistical power [36]. In this procedure, first, a fully specified model is estimated. Then, the independent variable with the highest *p*-value is removed and the model is re-estimated. The removal of variables is continued until there is no independent variable with a p-value higher than a certain cutoff (in our case this is.30). To support the robustness of our analyses, we additionally report the results of the full model in the S1 Appendix. Bivariate association tests (Pearson and Spearman Rank-Order correlations) also presented in the S1 Appendix.

While the initial selection of variables was based on theoretical considerations and expected relevant controls, the reported levels of significance should not be over-emphasized, given the relatively high number of candidate variables and risk of model overfitting. The reported findings are to be understood as an exploration of risk factors, and do not serve as hypothesis tests.

## Results

### Changes in social networks

**Fig 2:**
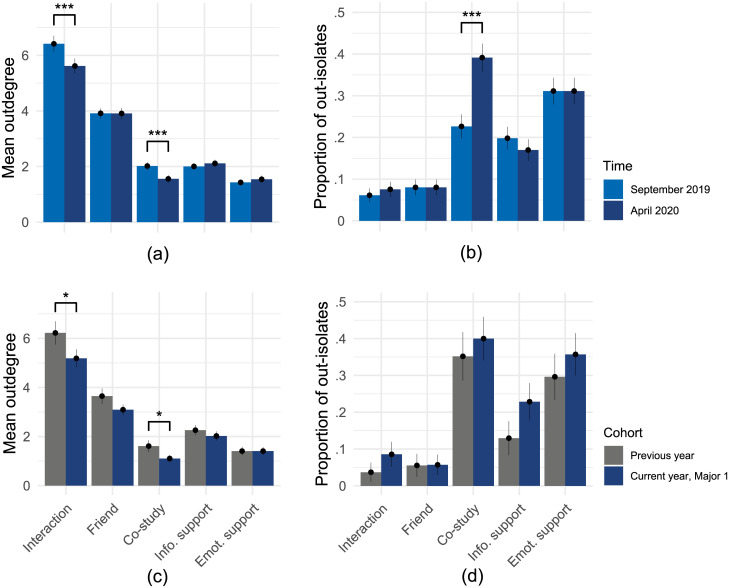
Numbers of reported ties and isolation. Students reported fewer pleasant interactions and fewer studying partners during lockdown than prior (within-subjects comparisons, panel a and b). Comparing the subset from one major to the previous year’s cohort of that major at the same time one year earlier (between-cohort comparison, panel c and d), outdegrees in the same two networks were significantly lower. Lines indicate one standard error above and below the mean estimate. *** *p* < .001; * *p* < .05.

Fig 2 presents a test of Hypotheses 1a and 1b. It indicates that social networks between students changed during the COVID-19 crisis. Panel a compares the average number of nominations (outdegree) across five self-reported networks (pleasant interaction, friendship, co-studying, informational support, emotional support) in September 2019 (prior to the COVID-19 lockdown) and April 2020 (at the time of the lockdown). Students reported on average 0.80 fewer interaction partners and 0.46 fewer study partners. These differences are found to be significant using one-sided, paired t-tests (Interaction: *t*(211) = 4.15, *p* < .001, *d* = .20; Co-study: *t*(211) = 3.60, *p* < .001, *d* = .24) among students from the current-year cohort who participated in both survey waves (*N* = 212). Hence, with regards to pleasant interaction and co-study networks, we find support for Hypothesis 1a (students nominate fewer fellow students). Friendship networks appeared to remain stable, while informational support and emotional support nominations slightly increased. Panel b shows the proportion of students who did not name a single contact in each of the networks (out-isolates). The proportion is significantly higher in the study network. Instead of 22.6% of the students, 39.2% now report not to have a study partner (McNemar’s *χ*^2^(1) = 21.81, *p* < .001, *OR* = 4.89). Hypothesis 1b (more students are socially isolated) is thus supported only for the co-study network. The network comparisons indicate that functional social networks between students (interaction and studying) decreased in density, while friendship and support networks remained stable.

Panel c and d of Fig 2 show the results of comparisons between the social networks of those students in Major I (*N* = 86) to the students from the same study program in the previous year (*N* = 54). In both cases the data were collected in April of the third study year, once in 2019 and once in 2020 (during the COVID-19 crisis). The direction is similar to the within-cohort comparison: Students in the semester affected by COVID-19 reported less contacts in each network, except for emotional support (Panel c), and were more likely to indicate that they were isolated, thus did not have a single contact in each of the five social networks (Panel D). The differences between the two cohorts reflect similar patterns of differences. Pleasant interaction and co-studying networks were significantly lower in outdegree, but no significant differences were found in the proportion of outisolates (using one-sided, independent-sample t-tests of lower outdegree in the current year compared to previous, Panel c; Interaction: *t*(112.07) = 1.73, *p* = .043, *d* = .30; Friend: *t*(101.23) = 1.46, *p* = .074, *d* = .26; Co-study: *t*(89.09) = 1.80, *p* = .038, *d* = .33; Info. support: *t*(121.45) = 0.85, *p* = .199, *d* = .14; Emot. support: *t*(125.08) = 0.00, *p* = .499, *d* = .00; and one-sided, independent-sample *z*-tests of greater proportion of isolates in the current-year cohort in Panel d; Interaction: *χ*^2^(1) = 1.74, *p* = .093, *d* = .28; Friend: *χ*^2^(1) = 0.23, *p* = .316, *d* = .14; Co-study: *χ*^2^(1) = 0.37, *p* = .271, *d* = .14; Info. support: *χ*^2^(1) = 1.64, *p* = .100, *d* = .26; Emot. support: *χ*^2^(1) = 0.36, *p* = .275, *d* = .14).

**Fig 3:**
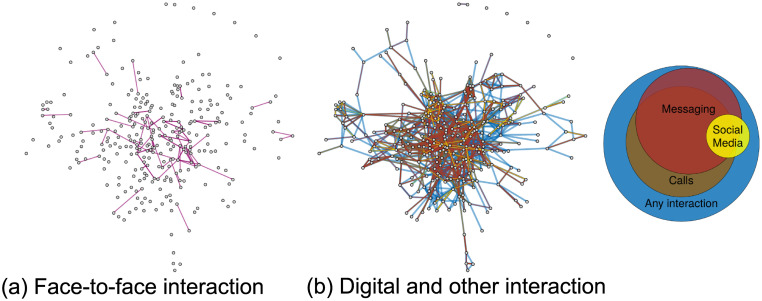
Social interaction networks between students during the COVID-19 crisis (April 2020). (**a**) Few face-to-face interactions were reported (pink ties). (**b**) Interaction networks through digital communication technologies were dense (social media: yellow ties, messaging apps: red, video and voice calls: brown, other pleasant interaction: blue). The Venn diagram indicates relative density and overlap between networks. Data from the current-year cohort, Major II (*N* = 294. This includes, in addition to all respondents to the April 2020 survey, all students enrolled in Major II who were nominated as connections by those who filled out the survey).

Fig 3 visualizes the reported interaction networks between students at the time of the COVID-19 crisis. As a consequence of the university lockdown and Swiss policies on social distancing, only few face-to-face interactions were reported by students (Panel a). Yet, students had pleasant interactions in general (*M*_*Interaction*_ = 5.62 ties, see Fig 2, Panel a). They reported to interact through text messaging, voice and video calls, and through social media (*M*_*Messaging*_ = 2.81;*M*_*Calls*_ = 2.94, *M*_*Social Media*_ = 0.41 ties). The union of these networks is indicated by blue ties (any interaction). Messaging, calls and social media ties are shown in red, green and yellow, respectively. The relative network density and overlap between them is shown in a Venn diagram in Fig 3. The interaction networks were overlapping, but not identical. 85% of the call ties and 90% of the messaging ties were reported as pleasant interactions. 64% of the messaging ties were also reported as call ties, and 70% of the call ties as messaging ties. Seventy-five percent of the physical interactions were overlapping with any of the digital communication networks. Additional Venn diagrams showing these overlaps can be found in the S1 Appendix. It appears that while physical interactions were rarely possible, students conducted their social interactions largely in the digital realm. Overall, however, the number of reported interactions dropped, as reported above. Additional network visualizations that illustrate tie change and social isolation in the co-study and social support networks are provided in the S1 Appendix.

**Fig 4:**
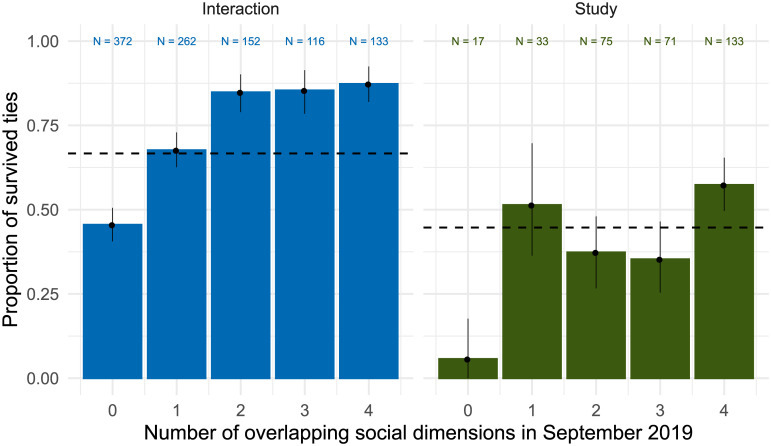
Proportion of stable (a) interaction and (b) co-study ties since September 2019 by the number of existing other network dimensions in September 2019. Dashed horizontal lines represents the network’s proportion of stable ties. Error bars indicate 95% bootstrapped Confidence Intervals.

Fig 4 presents a test on Hypothesis 2, suggesting that weaker, one-dimensional ties (i.e., zero overlapping social dimensions in September 2019) were less stable than stronger ties with more overlapping dimensions. It reports the empirical probability of interaction and studying ties to survive, thus to be reported in April 2020 after being reported in September 2019. Panel a shows that while in general 66.6% of the interaction ties survived (dashed line), the survival rate was only 45.4% for ties that did not coincide with any other of the four network relations (friendship, studying, informational, or emotional support). When interaction was reported with two to four of these networks in September 2019, the tie survival rate was between 84.9 and 87.2%. The pattern is similar in Panel b, which compares survival rates of co-studying ties. Ties that did not coincide with interaction, friendship, informational, or emotional support only had a 5.9% survival rate, while ties that overlapped with all four dimensions survived in 57.1% of the cases. The average survival rate was 44.7%.

### Changes in mental health

Here, we first describe how students rated a set of COVID-19 related stressors that specifically ask whether they feel more or less strained by a particular factor since the COVID-19 crisis. Then, we describe within-person and between-cohort comparisons of mental health changes with regards to depressive symptoms, anxiety, stress, and loneliness.

**Fig 5:**
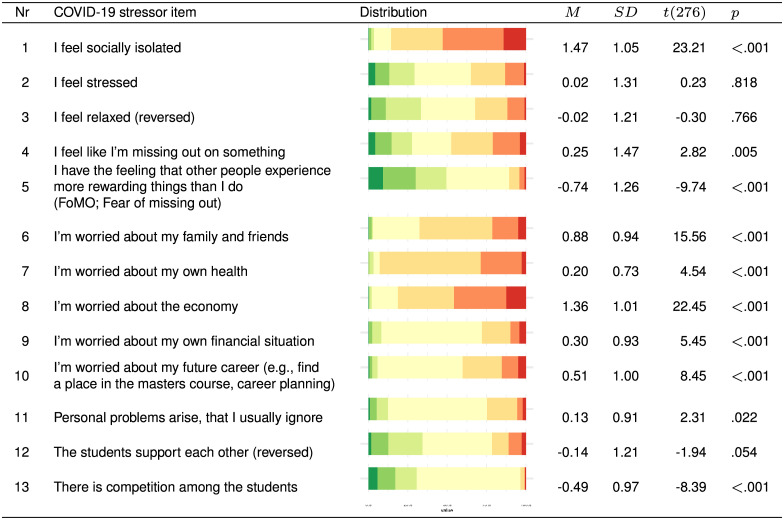
Specific COVID-19 stressor items and their distributions. *N* = 277, all current-year, April 2020 participants. Frequency distributions and t-test results (*H*_0_: *M* = 0) of each COVID-19-related stressor item. Rated on a 7-point scale (green = much less than before (-3), yellow = neutral (0), red = much more than before (+3)).

Fig 5 shows the distributions and means of responses on thirteen COVID-19-related stressors. The following effects are evaluated with one-sample, two-sided t-tests (*H*_0_: *M* = 0) as reported in Fig 5. Students felt significantly more socially isolated, slightly more that they were missing out on something, more worried about family and friends, slightly more worried about their own health, more worried about the economy, slightly more worried about their financial situation, more worried about their future career, and more affected by personal problems that were usually ignored. The students reported significantly less worries that others have more rewarding experiences (Fear of Missing Out; FoMO) and perceived less competition among the students. Students on average did not report differences with regards to stress, being relaxed, or perceived support among the students.

**Fig 6:**
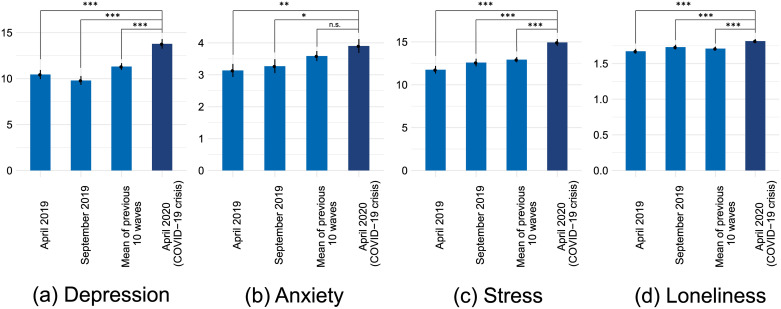
Mean scores and standard errors of the (a) depression, (b) anxiety, (c) stress, and (d) loneliness scale by measurement time-point (x-axis). The scales theoretically ranged between 0–60 (a), 0–21 (b), 0–40 (c), and 1–4 (d). The significance levels of paired t-tests of previous time-points with the COVID-19 wave (April 2020) are indicated by asterisks, *** *p* < .001; ** *p* < .01; * *p* < .05; n.s. = not significant.

Fig 6 shows the mean scores and standard errors of the four mental health markers (depression, anxiety, stress, loneliness) of the April 2020 wave (dark blue) in comparison with assessments of the same individuals of previous waves (April 2019, September 2019, and the person mean of ten waves assessed between September 2017 and September 2019; light blue). Paired t-tests indicate that since September 2019 the students became more depressed (*M*_diff_ = 4.44, *SE* = 0.50, *t*(208) = 8.89, *p* < .001, *d* = .34), slightly more anxious (*M*_diff_ = 0.60, *SE* = 0.24, *t*(208) = 2.47, *p* = .014, *d* = .10), more stressed (*M*_diff_ = 2.67, *SE* = 0.40, *t*(208) = 6.64, *p* < .001, *d* = .23), and more lonely (*M*_diff_ = 0.13, *SE* = 0.02, *t*(208) = 5.59, *p* < .001, *d* = .07). Similar negative trends were also observed for comparisons with data collected one year earlier (April 2019) and the person mean of the past ten waves (see Fig 6). Only the comparison of the April 2020 anxiety score with the person mean of past waves was not significant (*M* = 0.33, *SE* = 0.18, *t*(267) = 1.91, *p* = .057, *d* = .10). Overall, these within-person comparisons indicate that students on average report lower levels of mental health during the COVID-19 crisis than before the crisis.

We further compared the April 2020 mental health scores all Major I respondents of the current year (*N* = 83) to those of a cohort of students assessed a year earlier of the same study major (_=_ 54). The S1 Appendix compares the mean values of the two cohorts. In t-tests no significant differences were found with regards to depression (*M*_*April*2020_ = 13.77, *M*_*April*2019_ = 13.07, *t*(126) = 0.56, *p* = .578, *d* = .10), anxiety (*M*_*April*2020_ = 4.11, *M*_*April*2019_ = 4.41, *t*(126) = −0.52, *p* = .606, *d* = − .09), stress (*M*_*April*2020_ = 15.12, *M*_*April*2019_ = 14.76, *t*(126) = 0.31, *p* = .757, *d* = .05), and loneliness (*M*_*April*2020_ = 1.88, *M*_*April*2019_ = 1.84, *t*(126) = 0.45, *p* = .652, *d* = .08).

To investigate the comparability of the two cohorts, we explored trends in cohort-level mental health trajectories over the past two years. The S1 Appendix shows the mean trajectories and 95% confidence intervals of different mental health measures for those waves that were assessed at the same time with regards to the study progress. In most waves, the two cohorts did not differ significantly from one another (i.e., overlapping 95% Confidence Intervals). Only with regards to stress there appears to be a difference: the previous year’s cohort seems to be more stressed in general. The trends seem aligned through time, indicating seasonal and study-related factors that affect both cohorts. However, it appears that depression, stress, and loneliness (but not anxiety) at the time of the COVID-19 crisis worsen above the general trend, and that the co-studying outdegree (but not the interaction outdegree) decreases more than the general trend. These differences, however, are not statistically significant, as indicated by overlapping confidence intervals.

In sum, the descriptive results of the COVID-19 items support the expectation that some aspects of students mental health decrease (e.g., social isolation, general worries) while other stressors (e.g., Fear of Missing Out, student competition) are reduced during the COVID-19 crisis, as proposed in Hypothesis 3. The overall levels of mental health in comparison with the September 2019 wave show a clear decreasing trend. It is possible that these trends are due in part to factors unrelated to the COVID-19 crisis, as no significant differences with regards to mental health are found in a between-cohort comparisons, conducted on a smaller sub population.

### Explaining changes in mental health (explorative analysis)

Here, we describe the results of regression analyses on the *change* of mental health dimensions between September 2019 and April 2020 using a variety of explanatory variables including COVID-19-specific stressors, social networks between students, personal social networks, and demographic variables.

Students who participated in the September 2019 and April 2020 surveys reported an average increase in depressive symptoms of 4.70 (*SD* = 7.21, total scale range 0-60), an increase in anxiety of 0.75 (*SD* = 3.47, total scale range 0-21), an increase in stress of 2.76 (*SD* = 5.78, total scale range 0-40), and an increase in loneliness of 0.14 (*SD* = 0.33, total scale range 1-4).

Table 2 shows the results of backwards-selected regression models predicting these differences. Estimates of the full models as well as bivariate correlations of all independent variables with the dependent variables are reported in the S1 Appendix. For reasons of parsimony, in the following, mainly the significant predictors are discussed. It should be noted that this is an exploratory analysis, not intended to provide evidence for particular hypotheses. The results are discussed by each of the four predictor categories: the COVID-19 related stressors described above, measures of social integration in the social networks between students, measures of personal network integration, and demographics variables.

**Table 2:** Results of backwards-selected regression models predicting changes in depression, anxiety, stress, and loneliness between September 2019 and April 2020.

Variable	Depression change			Anxiety change			Stress change			Loneliness change		
	*b*	[lower CI, upper CI]	*β*	*b*	[lower CI, upper CI]	*β*	*b*	[lower CI, upper CI]	*β*	*b*	[lower CI, upper CI]	*β*
Intercept	1.26	[-2.13, 4.65]		0.55	[-0.22, 1.33]		4.93 ***	[2.69, 7.17]		0.53 ***	[0.34, 0.72]	
*COVID-19 items*												
Feeling socially isolated	0.60	[-0.42, 1.62]	0.08				0.53	[-0.24, 1.29]	0.10			
Feeling of missing out												
FoMO	0.52	[-0.31, 1.36]	0.09				-0.59	[-1.18, 0.01]	-0.13			
Worries about family and friends	1.54 **	[0.40, 2.67]	0.20	0.36	[-0.07, 0.79]	0.10	0.96 *	[0.10, 1.83]	0.16	0.05	[-0.00, 0.10]	0.14
Worries about own health							-0.81	[-1.97, 0.36]	-0.10	0.04	[-0.03, 0.12]	0.09
Worries about economics										0.04	[-0.01, 0.08]	0.10
Worries about own financial situation												
Worries about future career				0.45 *	[0.05, 0.84]	0.13	1.36 ***	[0.63, 2.09]	0.24			
Problems usually suppressed	4.10 ***	[2.75, 5.44]	0.44	1.49 ***	[1.03, 1.96]	0.38	0.93 *	[0.01, 1.85]	0.14	0.05	[-0.01, 0.10]	0.12
Student support	-0.52	[-1.37, 0.33]	-0.08				-0.39	[-1.00, 0.21]	-0.08			
Student competition										0.04	[-0.01, 0.09]	0.11
Any COVID-19 symptoms				0.71	[-0.14, 1.55]	0.10						
Someone in risk group												
Strict adherence (ref. not strict)	1.85	[-0.26, 3.96]	0.12	0.60	[-0.22, 1.42]	0.08						
*Student Social Networks*												
Outisolate friendship				-2.46 *	[-4.57, -0.36]	-0.16				0.27 *	[0.01, 0.53]	0.17
Outisolate pleasant interaction				2.22 *	[0.09, 4.35]	0.13	2.90	[-0.82, 6.61]	0.10	-0.14	[-0.39, 0.10]	-0.09
Outisolate emotional support							-1.32	[-3.05, 0.42]	-0.10			
Outisolate informational support				2.38 ***	[1.08, 3.69]	0.23				0.14	[-0.01, 0.29]	0.14
Outisolate co-study										-0.08	[-0.18, 0.03]	-0.11
*Personal Network*												
Network size (centered)	-0.42	[-0.85, 0.01]	-0.14	-0.13	[-0.30, 0.04]	-0.09	-0.17	[-0.49, 0.15]	-0.07	-0.03 *	[-0.05, -0.01]	-0.19
Isolated household	3.19 *	[0.16, 6.23]	0.15							-0.11	[-0.26, 0.03]	-0.11
Minimal physical contact (centered)				-0.33 *	[-0.57, -0.08]	-0.15	-0.49	[-0.98, 0.01]	-0.13			
Mean emotional support (centered)	-1.14 *	[-2.21, -0.07]	-0.16	-0.24	[-0.66, 0.18]	-0.07				-0.07 **	[-0.12, -0.02]	-0.21
*Demographics*												
Study Major (ref. Major 1)	2.93 *	[0.71, 5.15]	0.18									
Single (ref. in relationships)							1.23	[-0.37, 2.83]	0.11	0.10 *	[0.00, 0.20]	0.16
Extraversion (centered)	-1.03	[-2.54, 0.48]	-0.10									
Being female (ref. male)	3.05 *	[0.65, 5.46]	0.18	1.32 **	[0.40, 2.24]	0.17	2.04 *	[0.20, 3.88]	0.15	0.15 **	[0.04, 0.26]	0.20
Non Swiss							-1.38	[-3.52, 0.75]	-0.08			
*Controls*												
Depression previous wave	-0.32 ***	[-0.50, -0.14]	-0.26									
Anxiety previous wave				-0.58 ***	[-0.72, -0.45]	-0.52						
Stress previous wave							-0.45 ***	[-0.58, -0.33]	-0.48			
Loneliness previous wave										-0.34 ***	[-0.45, -0.23]	-0.50
R2	0.42			0.48			0.40			0.29		

N = 144–178 (list-wise selection).

* *p* < .05,

** *p* < .01,

*** *p* < .001.

*b* = unstandardized regression coefficient, *β* = standardized regression coefficient, CI = 95% Confidence Interval.

The results on COVID-19 related stressors indicate that students who worry more about their family and friends are more likely to become more depressed (*b* = 1.54, *t*(130) = 2.68, *p* = .008) and more stressed (*b* = 0.96, *t*(159) = 2.19, *p* = .030). Worries about one’s future career contributed to higher levels of anxiousness (*b* = 0.45, *t*(164) = 2.22, *p* = .027) and stress (*b* = 1.36, *t*(159) = 3.69, *p* < .001). The presence of personal problems that were usually suppressed was a strong positive predictor of an increase in depression (*b* = 4.10, *t*(130) = 6.03, *p* < .001), anxiety (*b* = 1.49, *t*(164) = 6.31, *p* < .001), and stress (*b* = 0.93, *t*(159) = 2.01, *p* = .047). No other COVID-19 related stressors was associated with changes in mental health. Although social isolation and worries about the economy increased since the COVID-19 crisis (see Fig 5), these factors did not predict changes in mental health in this multivariate regression model (for details see Table 2).

The results regarding the effects of being isolated in social networks between students on mental health change are found next in Table 2. Social isolation is associated with changes in anxiety: Being an out-isolate (i.e., having no outgoing ties) in the friendship network in April 2020 was predictive of lower levels of anxiety (*b* = −2.46, *t*(164) = −2.31, *p* = .022), while being an out-isolate in the interaction network (*b* = 2.22, *t*(164) = 2.06, *p* = .041) and the informational support network (*b* = 2.38, *t*(164) = 3.60, *p* < .001) was predictive of higher levels of anxiety. It should be noted that the subset of students who were isolated only in the friendship network but not in the interaction and information support network is very small (*N* = 4). Thus, for most students isolation in student social networks is associated with an increase in anxiety. Further, being an out-isolate in the friendship network is associated with increasing loneliness trajectories (*b* = 0.27, *t*(161) = 2.05, *p* = .042).

Characteristics of personal networks assessed during the COVID-19 crisis are also associated with changes in mental health. Individuals with smaller personal networks appear to become more lonely (*b* = −0.27, *t*(161) = −2.48, *p* = .014). Those students who live alone (i.e., are isolated from their personal network in their household) are more likely to report an increase in depressive symptoms (*b* = 3.19, *t*(130) = 2.08, *p* = .039), and those who interact less with individuals in their personal network tend to be more anxious (*b* = −0.33, *t*(164) = −2.60, *p* = .010). We further find that those with more emotional support were less depressed (*b* = −1.14, *t*(130) = −2.10, *p* = .038) and less lonely (*b* = −0.07, *t*(161) = −2.70, *p* = .008).

Demographic variables are also associated with mental health trajectories. Individuals not in a romantic relationship were more likely to become more lonely (*b* = 0.10, *t*(161) = 2.05, *p* = .042). Female students are found to be more likely to become more depressed (*b* = 3.05, *t*(130) = 2.51, *p* = .013), anxious (*b* = 2.04, *t*(164) = 2.20, *p* = .030), stressed (*b* = 1.32, *t*(159) = 2.82, *p* = .005), and lonely (*b* = 0.15, *t*(161) = 2.68, *p* = .008) in these regression models. In bivariate tests, gender was not significantly associated with changes in mental health (for details see the S1 Appendix). This may indicate a suppressing mediating effect of different forms of social integration on females’ mental health. In general, female students tend to be better socially integrated and to report more sources of support than male students (for details, see the S1 Appendix). Previous work indeed suggests that women rely more on social networks than men [37]. Hence, female students lacking such support networks at the time of crisis may be worse off than male students.

## Discussion

In this study, we investigate the change in social networks (social interactions, social support, study collaboration, friendship) and mental health (depression, anxiety, loneliness, stress) of students in two undergraduate programs at a Swiss university at the time of the COVID-19 crisis. At this point, the university had been under lockdown for about two weeks. The Swiss government had further implemented a number of social distancing measures and individuals were demanded to stay at home. We compare measures on social networks and mental health to (i) those of the same students seven months earlier (prior to the COVID-19 outbreak; within-person comparison) and (ii) those collected in a different student community with the same study progress (final undergraduate year) at the same university and in the same study program exactly one year earlier (between-cohort comparison). Finally, in exploratory analyses, we identify a number of individual and social factors associated with changes in mental health in this period.

In within-person comparisons of social networks and in line with *H1*, we observe that students nominated fewer others in social interaction networks and co-study networks and were more likely to be isolated in co-study networks. Friendship and social support networks did not change significantly. In between-cohort comparisons of social networks, we similarly observe significantly lower connectivity (outdegree) in pleasant interaction and co-study networks, but no significant differences in proportion of socially isolated individuals. Stronger social ties, those characterized by overlap with other dimensions half a year earlier, were more likely to be maintained during the social distancing phase, in line with *H2*. Overall, most students remained well-integrated after two weeks of physical distancing and it appeared that while face-to-face interaction was unattainable, digital communication was very common. However, the increasing number of isolated individuals in two of the networks (interaction and studying) suggest that some individuals might be at higher risk of facing negative social consequences.

In within-person comparisons of mental health dimensions, we find that students were on average more depressed, slightly more anxious, more stressed, and felt more lonely than half a year earlier. While measures of mental health suggest a decline, some students reported that the crisis situation affected their lives positively with regards to Fear of Missing Out and competition among the students. These findings are in line with *H3*, that students perceived both positive and negative changes due to the situation. In between-cohort comparisons of mental health measures no significant differences were observed, although the trends appear similar.

Finally, we identify factors affecting negative and positive mental health progressions. In particular females were more likely to be affected negatively by the situation when holding other factors constant, but often they could rely on denser support networks potentially helping them to buffer the negative effects of the crisis. Female students were a minority group in all student cohorts, which might contribute to their potentially higher vulnerability in our sample. Further, it appears that a worsening of mental health measures was associated with more worries about one’s family and friends, more worries about the future career, being faced with problems usually suppressed, living alone, and less contact and support from the personal network. These findings complement earlier empirical research discussing the importance of social networks on well-being and mental health [18, 38], and gendered differences in the size of social support networks, e.g., [37]. They are further in line with studies showing those individuals who worry more [39], who are more socially isolated [40], and receive less social support [41, 42] are more likely to develop mental health problems. Those individuals are also more at risk for serious health conditions [42, 43].

A limitation of this study is that the observational research design does not allow us to make causal claims. Some of the changes may be related to general trends within the study program. Students were in the final year of their undergraduate studies and might feel more strained because they have to pass final exams and get active on the job market. While the comparison to a cohort of students in the previous year indicated no mental-health differences, this comparison is limited due to differences in demographic distributions and cohort-related factors. However, the fact that COVID-19-specific stressors were associated with change in mental health, gives us confidence that indeed the COVID-19 crisis had a negative impact on the social networks and mental health of the students under lockdown.

Another limitation concerns the application of the backwards selection procedure for regression models explaining changes in mental health. Selecting on statistical associations is a data-driven approach which may lead to non-replicable results [44]. The exploratory findings were not guided entirely by theoretical expectations and might be specific to the empirical context of the current study. Furthermore, this lead to a large number of statistical tests, and focusing on any single test in isolation may lead to misjudging the certainty of the results. However, we hope that the reported descriptive associations encourage replications of the research design in different settings and further theoretical elaboration. At the time of writing, the COVID-19 crisis is still unfolding, which further limits the generalizability of our findings.

### Conclusion

Students’ social networks and mental health trajectories cannot be understood independently of each other [45]. It is therefore important to study how the COVID-19 crisis and related measures affect the social networks and mental health of students. It appears that the university lockdown and social distancing measures negatively affect the social integration of some individuals, partly leaving them isolated, while in fact more social support might be needed to cope with the additional stress factors.

Some students might be at higher risk of social isolation and the development of mental health problems during the COVID-19 crisis. In particular, when they live by themselves, have less direct contact to close family members and friends, receive less social support, and have a weaker integration in the social networks of students. Female students (who were the minority group in the student populations) appeared to be at higher risk of facing negative mental health consequences. These observations as well as those of similar studies are crucial to develop targeted interventions to support students who are potentially at risk. These could include digital forms of study groups, peer group sessions, mentoring, and psychological counseling [46].

Our findings may further inform ongoing efforts of universities around the world to develop new hybrid teaching strategies for the coming academic years. These will increasingly have to rely on online learning as a complement to traditional classroom teaching. It appears that students should receive opportunities to interact and socialize in informal social settings. This could matter more for newly enrolled students who did not have the chance to form social ties with others, yet. If this is not possible in-person due to COVID-19 constraints, university managers and teachers should consider the development of online events and the use of online platforms to support the development of social ties between students. Friendship, interaction, social support, and studying with others have been argued to impact their well-being and academic success, but they often require meeting opportunities and informal settings to develop [5, 13].

The flattening of the infection curve during the COVID-19 pandemic requires strict public health measures, such as social distancing, closure of public institutions, and a reduction of social life. But when implementing such measures nationally and at universities, it is important to consider and counteract potential negative effects on individuals’ social networks and mental health.

## Supporting information

S1 Appendix Survey items, network descriptives, visualizations, regression models, variable correlations. (PDF)
