# Supplementary material for: Students under lockdown: Comparisons of students’ social networks and mental health before and during the COVID-19 crisis in Switzerland
Source: PLoS One. 2020 Jul 23;15(7):e0236337. doi: 10.1371/journal.pone.0236337 (PMC7377438; doi:10.1371/journal.pone.0236337)
Supplement: S1 Appendix — (PDF) [file pone.0236337.s001.pdf]

Supplementary Information: Students under lockdown: Comparisons of students' social networks and mental health before and during the COVID-19 crisis in Switzerland

Timon Elmer[1], Kieran Mepham[1], & Christoph Stadtfeld[1]
[1]Chair of Social Networks, ETH Zürich, Switzerland

July 10, 2020

Contents

1 Survey items **2**
1.1 Social Network Items 2
1.2 Personal network questionnaires 2
1.2.1 Introductory text for personal network questions 2
1.2.2 Item on the role of each named member of the personal network 3
1.2.3 Items on the mode of contact for each named member of the personal network 3
1.2.4 Items on the frequency of each mode of contact 3
1.3 COVID-19-specific item texts 3
1.3.1 Own symptoms COVID-19 3
1.3.2 Item battery on concerns relative to pre-COVID-19 4

2 Network reciprocity rates **4**

3 Additional network and mental health visualizations **6**
3.1 Overlap between interaction networks 6
3.2 Change in studying and social support networks 6
3.3 Between-cohort mental health comparison 6
3.4 Between-cohort trajectory comparison 6

4 Full regression models **6**

5 Variable correlations **12**

1 Survey items

Where answer categories are not given, these can be found in the main text in the relevant section of the measures [1]. Text between squared brackets ([]) gives further information to the reader, but was not shown to survey respondents. Additional details about measures are provided in [2].

1.1 Social Network Items

Full item texts referenced in the main text are in Table 1.

Table 1: Short item names and text of social network questions

Item name	Question text
Interaction	With whom do you have pleasant interactions?
Friend	Which of your fellow students would you call a friend?
Co-study	With whom do you regularly spend time studying together? Working on a joint project or homework and revising lecture materials together both count.
Info. support	Whom can you count on when you need good advice or specific information?
Emot. support	Whom can you count on when you're feeling bad and you need emotional support (e.g., consolation, empathy and encouragement)?
Call	With whom do you interact by telephony (by telephone, Skype, FaceTime, etc.)?
Phys. comm.	With whom do you interact in-person (you are physically together)?
Messaging	With which of your fellow students do you regularly communicate over messaging apps? (for instance Whatsapp, Signal, SMS)
Social media	With which of your fellow students do you regularly communicate over social media? (excluding messaging apps)

1.2 Personal network questionnaires

1.2.1 Introductory text for personal network questions

Please name the people who are important to you during the COVID-19 crisis. These could be co-students as well as people from outside your studies, and may include people with whom you do not get along. These could be, for example, family members, your partner, colleagues, housemates, etc. [10 open text fields follow]

1.2.2 Item on the role of each named member of the personal network

To me, this person is... [multiple answers possible]

- family
- a romantic partner
- a friend
- a roommate
- a work colleague
- other

1.2.3 Items on the mode of contact for each named member of the personal network

I have the following contact with this person... [multiple answers possible]

- In the same household
- outside the household
- by telephone (audio only)
- by Skype/FaceTime or similar
- Chat-messaging (e.g. Whatsapp, Signal)
- Other [open text]

1.2.4 Items on the frequency of each mode of contact

- How often do you speak to this person?
- How often do you have in-person contact with this person? (you are physically together)
- How often do you have telephone contact with this person? (e.g. by telephone, Skype, Facetime, etc.)
- How often do you communicate with this person over messaging apps? (e.g. Whatsapp, Signal, SMS)

1.3 COVID-19-specific item texts

1.3.1 Own symptoms COVID-19

Have you had any of the following symptoms, which have been connected to COVID-19? [multiple answers possible]

- Fever
- Coughing
- Decreased sense of taste
- Shortness of breath

- Sore throat
- Muscle ache
- Other [open text]

1.3.2 Item battery on concerns relative to pre-COVID-19

All responses were measured on a 7pt scale from -3 (much less than before) through 0 (equally) to 3 (much more than before).

- I feel socially isolated
- I feel stressed
- I feel relaxed
- I feel that I am missing out on things
- I have the feeling that others are having more rewarding experiences than me (FoMO; fear of missing out)
- I am worried about my family and friends
- I am worried about my own health
- I am worried about the economy
- I am worried about my own financial situation
- I am worried about my future career (e.g. Master's program, job situation)
- Personal problems arise, that I usually ignore
- Students compete with each other
- Students support each another

2 Network reciprocity rates

Table 2 provides an overview of reciprocity rates in six networks measured within the student cohort. Rates are calculated only for dyads where both individuals participated in the survey.

Table 2: Reciprocity rates of cohort networks amongst participants

	Interaction	Friend	Co-study	Info. support	Emot. support	Msg. comm.
M I, curr, Sept 2019	0.48	0.55	0.44	0.38	0.35	0.48
M I, curr., Apr 2020	0.45	0.53	0.30	0.31	0.29	0.40
M II, curr., Sept 2019	0.42	0.51	0.46	0.28	0.28	0.53
M II, curr., Apr 2020	0.39	0.47	0.35	0.35	0.39	0.50
M I, prev., Apr 2019	0.49	0.58	0.30	0.42	0.31	0.46

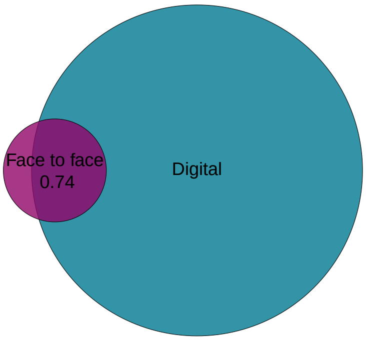

(a)

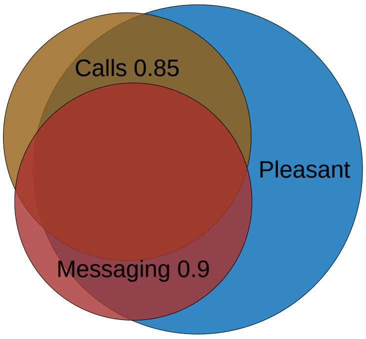

(b)

Figure 1: (**a**) 74% of the reported face-to-face interactions overlap with any type of digital interaction (calls, messages, social media), see Fig 2 in main text; (**b**) Calls and messaging ties are mostly also reported as pleasant interactions (85% and 90%, respectively, see Fig 2 in main text)

3 Additional network and mental health visualizations

3.1 Overlap between interaction networks

Figure 1 shows the relative size and overlap between different network measures, similar to Figure 2 in the main text.

3.2 Change in studying and social support networks

Figure 2 visualizes change in studying and support networks.

3.3 Between-cohort mental health comparison

Figure 3 shows the between-cohort mental health comparison.

3.4 Between-cohort trajectory comparison

Figure 4 illustrates mental health trajectories between the two age cohorts at comparable times during their studies. One cohort was affected by the COVID-19 pandemic, this other was not as they studied one year earlier.

4 Full regression models

Table 3 includes the full results of the backwards-selected regression models, reported in the main text.

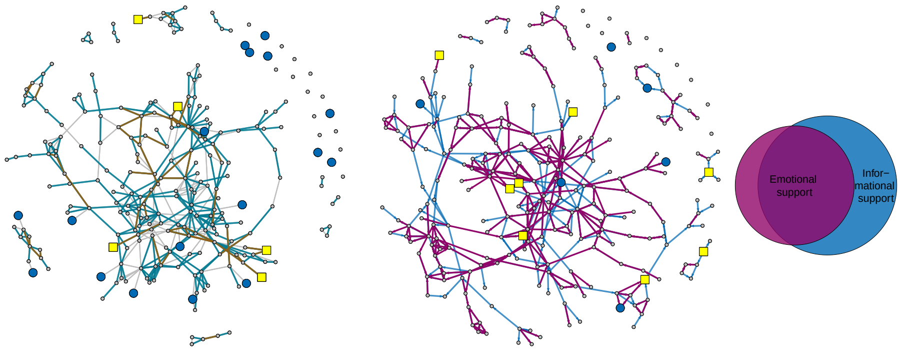

(a) (b)

Figure 2: (**a**) Reported studying ties. Newly created relationships are shown in brown, stable relationships in cyan, decayed relationships in gray. (**b**) Emotional support ties (pink) and informational support ties (blue). Newly isolated nodes are represented by larger blue circles (out-isolates in case of the support networks), newly integrated nodes by yellow squares. The Venn diagram indicates relative density and overlap between the networks. Data from the current-year cohort, Major II.

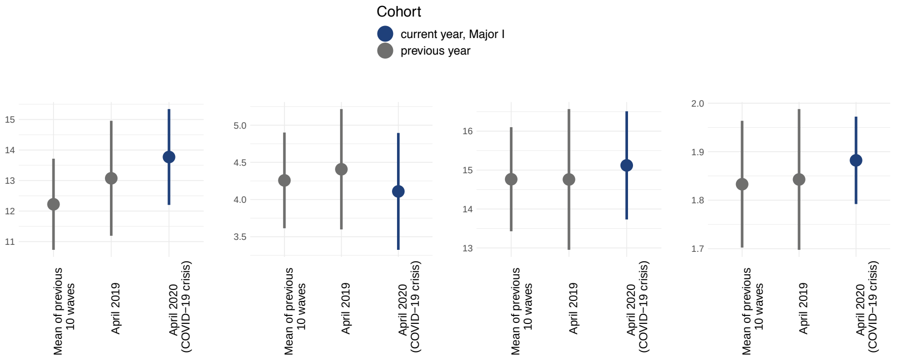

(a) depression (b) anxiety (c) stress (d) loneliness

Figure 3: Means and 95% Confidence Intervals of the (a) depression, (b) anxiety, (c) stress, and (d) loneliness scale by measurement time-point (x-axis) and cohort.

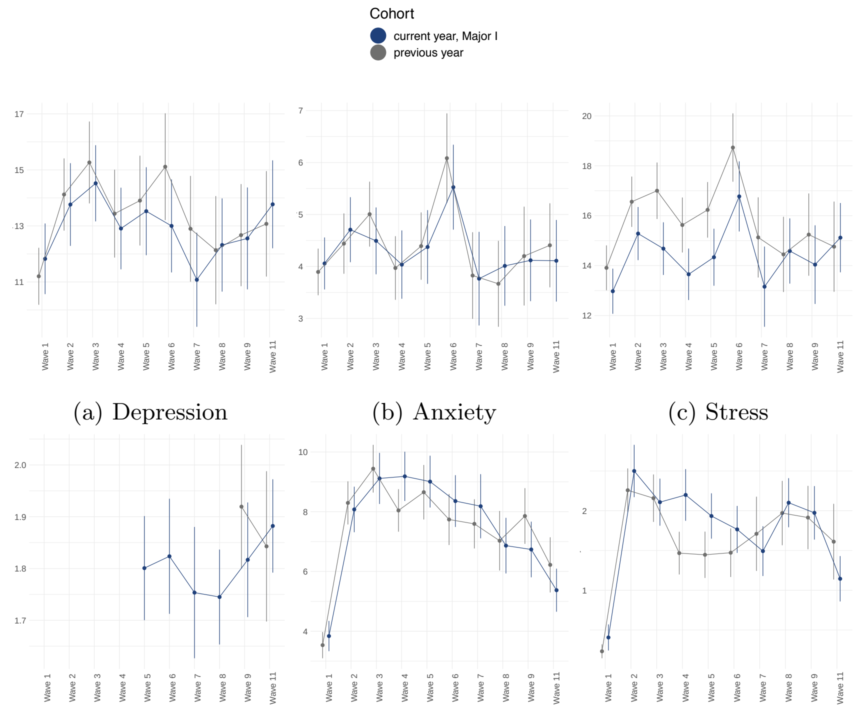

(a) Depression (b) Anxiety (c) Stress

(d) Loneliness (e) Interaction Outdegree (f) Co-study Outdegree

Figure 4: Trajectories of means and 95% Confidence Intervals of key measurements of the current year cohort Major 1 (blue) and the previous year cohort (gray). Wave 10 corresponds to the April 2020 wave of the current year cohort and April 2019 wave of the previous year. Wave 1 to 8 were collected between September 2017 and March 2019 (current year cohort) and September 2016 and March 2018 (previous year cohort), and correspond between cohorts with regards to the study-progress. Wave 9 was removed from this plot because the survey dates of the two cohorts did not overlap with respect to the semester-progress. Loneliness was not measured in all waves of all cohorts.

Table 3: Results of full regression models predicting changes in depression, anxiety, stress, and loneliness between September 2019 and April 2020

	Depression change				Anxiety change				Stress change				Loneliness change			
	b	[lower CI, upper CI]	β	VIF	b	[lower CI, upper CI]	β	VIF	b	[lower CI, upper CI]	β	VIF	b	[lower CI, upper CI]	β	VIF
Intercept	1.19	[-3.33, 5.70]			0.50	[-1.38, 2.38]			5.40**	[1.89, 8.92]			0.60***	[0.31, 0.90]		
COVID-19 items																
Feeling socially isolated	0.48	[-0.83, 1.79]	0.07	1.65	−0.14	[-0.70, 0.41]	−0.04	1.66	0.39	[-0.62, 1.40]	0.07	1.67	−0.03	[-0.09, 0.04]	−0.08	1.69
Feeling of missing out	−0.27	[-1.37, 0.84]	−0.05	2.07	0.06	[-0.38, 0.50]	0.02	1.89	−0.04	[-0.84, 0.76]	−0.01	1.86	0.03	[-0.02, 0.08]	0.11	1.88
FoMO	0.63	[-0.47, 1.73]	0.10	1.64	−0.13	[-0.56, 0.30]	−0.04	1.48	−0.95*	[-1.75, -0.15]	−0.20	1.43	0.01	[-0.04, 0.06]	0.04	1.44
Worries about family and friends	1.71*	[0.26, 3.16]	0.22	1.69	0.34	[-0.27, 0.94]	0.09	1.70	1.24*	[0.10, 2.37]	0.20	1.67	0.07	[-0.00, 0.14]	0.19	1.68
Worries about own health	−0.37	[-2.46, 1.72]	−0.03	1.75	−0.23	[-1.15, 0.69]	−0.04	1.79	−1.62	[-3.26, 0.02]	−0.18	1.85	0.02	[-0.08, 0.13]	0.04	1.78
Worries about economics	0.06	[-1.24, 1.36]	0.01	1.38	0.14	[-0.41, 0.69]	0.04	1.40	−0.80	[-1.82, 0.22]	−0.13	1.36	0.04	[-0.02, 0.10]	0.11	1.33
Worries about own financial situation	−0.24	[-1.65, 1.17]	−0.03	1.23	0.03	[-0.57, 0.63]	0.01	1.28	0.63	[-0.50, 1.77]	0.09	1.24	−0.04	[-0.11, 0.03]	−0.09	1.28
Worries about future career	0.34	[-0.85, 1.52]	0.05	1.37	0.25	[-0.26, 0.75]	0.07	1.35	1.18*	[0.26, 2.10]	0.21	1.35	0.00	[-0.05, 0.06]	0.01	1.37
Problems usually suppressed	4.19***	[2.64, 5.73]	0.46	1.42	1.77***	[1.15, 2.39]	0.42	1.36	1.39*	[0.27, 2.51]	0.20	1.39	0.04	[-0.03, 0.11]	0.11	1.39
Student support	−0.55	[-1.55, 0.45]	−0.09	1.33	0.14	[-0.27, 0.56]	0.05	1.28	−0.28	[-1.04, 0.48]	−0.06	1.26	0.00	[-0.05, 0.05]	−0.01	1.27
Student competition	0.32	[-0.87, 1.50]	0.04	1.24	−0.02	[-0.53, 0.49]	−0.01	1.25	0.18	[-0.76, 1.12]	0.03	1.21	0.03	[-0.03, 0.08]	0.08	1.23
Any COVID-19 symptoms	0.82	[-1.85, 3.49]	0.05	1.43	1.00	[-0.14, 2.13]	0.13	1.40	0.81	[-1.23, 2.86]	0.07	1.44	0.02	[-0.11, 0.14]	0.02	1.37
Someone in risk group	0.41	[-3.10, 3.92]	0.02	1.43	0.00	[-1.53, 1.53]	0.00	1.40	−0.49	[-3.21, 2.24]	−0.03	1.45	0.08	[-0.09, 0.25]	0.09	1.37
Strict adherence (ref. not strict)	1.73	[-0.68, 4.14]	0.11	1.25	1.01	[-0.02, 2.04]	0.14	1.25	−0.13	[-2.00, 1.75]	−0.01	1.25	0.04	[-0.08, 0.15]	0.05	1.27
Student Social Networks																
Outisolate friendship	0.99	[-6.18, 8.15]	0.02	1.61	−1.04	[-4.12, 2.04]	−0.05	1.59	2.24	[-3.36, 7.83]	0.07	1.59	0.37	[-0.02, 0.77]	0.18	1.63

Outisolate pleasant interaction	−0.14	[-6.21, 5.93]	0.00	1.15	2.40	[-0.24, 5.03]	0.12	1.11	2.60	[-2.60, 7.80]	0.07	1.16	−0.28	[-0.58, 0.02]	−0.15	1.16
Outisolate emotional support	−0.11	[-3.24, 3.03]	−0.01	1.64	−0.59	[-1.91, 0.74]	−0.07	1.62	−1.79	[-4.21, 0.62]	−0.13	1.62	−0.03	[-0.18, 0.12]	−0.04	1.62
Outisolate informational support	1.16	[-3.24, 5.55]	0.05	1.58	2.55**	[0.67, 4.43]	0.21	1.50	0.40	[-3.08, 3.87]	0.02	1.55	0.18	[-0.03, 0.40]	0.16	1.62
Outisolate co-study	−0.44	[-3.31, 2.44]	−0.03	1.71	−0.25	[-1.47, 0.96]	−0.03	1.69	−0.15	[-2.37, 2.07]	−0.01	1.69	−0.08	[-0.22, 0.06]	−0.11	1.74

(table continues on next page)

Table 3: Results of full regression models predicting changes in depression, anxiety, stress, and loneliness between September 2019 and April 2020 (continued)

Variable	Depression change				Anxiety change				Stress change				Loneliness change			
	b	[lower CI, upper CI]	β	VIF	b	[lower CI, upper CI]	β	VIF	b	[lower CI, upper CI]	β	VIF	b	[lower CI, upper CI]	β	VIF
Personal Network																
Network size (centered)	−0.48	[-1.02, 0.06]	−0.16	1.58	−0.12	[-0.36, 0.11]	−0.08	1.59	−0.25	[-0.68, 0.17]	−0.10	1.58	−0.02	[-0.05, 0.01]	−0.14	1.60
Isolated household	1.89	[-3.65, 7.43]	0.09	3.12	1.07	[-1.21, 3.35]	0.10	2.85	1.43	[-2.72, 5.57]	0.08	2.84	−0.15	[-0.41, 0.11]	−0.15	2.84
Minimal physical contact (centered)	−0.36	[-1.55, 0.83]	−0.08	3.17	−0.31	[-0.79, 0.17]	−0.14	2.90	−0.35	[-1.22, 0.52]	−0.09	2.91	0.00	[-0.06, 0.05]	−0.01	2.93
Mean emotional support (centered)	−1.03	[-2.37, 0.31]	−0.14	1.70	−0.21	[-0.78, 0.37]	−0.06	1.74	0.15	[-0.91, 1.20]	0.03	1.72	−0.07*	[-0.14, -0.01]	−0.22	1.83
Demographics																
Study Major (ref. Major 1)	2.71*	[0.07, 5.35]	0.17	1.39	0.25	[-0.89, 1.39]	0.03	1.36	0.22	[-1.83, 2.27]	0.02	1.41	−0.04	[-0.17, 0.08]	−0.06	1.37
Single (ref. in relationships)	1.00	[-1.70, 3.69]	0.07	1.66	0.03	[-1.13, 1.20]	0.00	1.68	1.59	[-0.52, 3.70]	0.14	1.70	0.07	[-0.07, 0.20]	0.10	1.66
Extraversion (centered)	−0.96	[-2.79, 0.87]	−0.09	1.55	−0.23	[-1.00, 0.53]	−0.05	1.50	−0.28	[-1.68, 1.13]	−0.03	1.48	0.00	[-0.10, 0.09]	−0.01	1.69
Being female (ref. male)	3.07*	[0.24, 5.90]	0.18	1.44	1.59*	[0.38, 2.80]	0.20	1.43	1.73	[-0.48, 3.93]	0.13	1.43	0.12	[-0.02, 0.26]	0.16	1.44
Non Swiss	−0.25	[-3.79, 3.29]	−0.01	1.34	−0.99	[-2.46, 0.49]	−0.10	1.35	−2.66	[-5.34, 0.01]	−0.16	1.35	−0.10	[-0.27, 0.07]	−0.11	1.36
Controls																
Depression previous wave	−0.33**	[-0.54, -0.13]	−0.27	1.37												
Anxiety previous wave					−0.64***	[-0.84, -0.43]	−0.46	1.38								
Stress previous wave									−0.41***	[-0.57, -0.24]	−0.40	1.41				
Loneliness previous wave													−0.33***	[-0.48, -0.18]	−0.47	1.90
R2	0.43				0.54				0.45				0.34			

Notes. N = 141–144 (list-wise selection). * $p < .05$,** $p < .01$,*** $p < .001$. b = unstandardized regression coefficient, β = standardized regression coefficient, CI = 95% Confidence Interval, VIF = variance inflation factor.

5 Variable correlations

Table 4 includes correlations between central variables of the analyses.

Table 4: Correlations of regression predictors and mental health changes

	Depression change	Stress change	Anxiety change	Loneliness change
Depression change				
Stress change	0.48***			
Anxiety change	0.61***	0.43***		
Loneliness change	0.23**	0.00	0.13	
COVID-19 items				
Feeling socially isolated	0.26***	0.23**	0.18*	0.00
Feeling of missing out	0.18*	0.00	0.05	0.09
FoMO	0.19*	−0.11	0.05	0.10
Worries about family and friends	0.17*	0.04	0.07	0.15*
Worries about own health	0.04	−0.08	0.00	0.08
Worries about economics	0.01	0.00	0.02	0.10
Worries about own financial situation	0.05	0.02	0.04	0.01
Worries about future career	0.19**	0.22**	0.17*	0.08
Problems usually suppressed	0.42***	0.13	0.34***	0.10
Student support	−0.13	−0.16*	−0.01	−0.04
Student competition	0.08	0.10	0.010	0.13
Any COVID-19 symptoms[1]	−0.06	−0.09	0.03	0.03
Someone in risk group[1]	0.07	0.04	0.03	−0.08
Strict adherence (ref. not strict)[1]	0.07	−0.03	0.09	0.04
Student Networks				
Out-isolate friendship[1]	0.08	0.00	0.01	0.08
Out-isolate pleasant interaction[1]	0.11	0.05	0.14	0.00
Out-isolate emotional support[1]	0.07	−0.10	−0.03	0.03
Out-isolate informational support[1]	0.13	0.02	0.15*	0.07
Out-isolate co-study[1]	0.04	−0.01	−0.01	−0.09

(table continues on next page)

Table 4: Correlations of regression predictors and mental health changes (continued)

	depression change	anxiety change	stress change	loneliness change
Personal Networks				
Network size (centered)	−0.02	0.06	−0.05	−0.02
Isolated household[1]	0.06	0.11	0.12	−0.09
Minimal physical contact (centered)	−0.1	−0.16*	−0.13	0.1
Mean emotional support (centered)	−0.1	0.04	0.02	0.01
Demographics				
Single (ref. in relationships)[1]	0.09	0.02	−0.01	−0.05
Extraversion (centered)	−0.05	0.01	0.03	0.15
Being female (ref. male[1])	0.05	0.14	0.09	0.13
Non Swiss[1]	−0.04	−0.04	−0.06	−0.09

Notes. $*p < .05$, $**p < .01$, $***p < .001$, [1] Spearman rank correlations

References

[1] Elmer T, Mepham K, Stadtfeld C. Students underlockdown: Comparisons of students' social networks and mental health before and during the COVID-19 crisis in Switzerland.

[2] Stadtfeld C, Vörös A, Elmer T, Boda Z, Raabe IJ. Integration in emerging social networks explains academic failure and success. Proceedings of the National Academy of Sciences. 2019;116(3):792–797.
